# Structure of the Low pH Conformation of Chandipura Virus G Reveals Important Features in the Evolution of the Vesiculovirus Glycoprotein

**DOI:** 10.1371/journal.ppat.1004756

**Published:** 2015-03-24

**Authors:** Eduard Baquero, Aurélie A. Albertini, Hélène Raux, Linda Buonocore, John K. Rose, Stéphane Bressanelli, Yves Gaudin

**Affiliations:** 1 Institute for Integrative Biology of the Cell (I2BC), CEA, CNRS, Université Paris-Sud, Gif-sur-Yvette, France; 2 Yale University School of Medicine, New Haven, Connecticut, United States of America; Harvard Medical School, UNITED STATES

## Abstract

Chandipura virus (CHAV), a member of the vesiculovirus genus, is an emerging human pathogen. As for other rhabdoviruses, CHAV entry into susceptible cells is mediated by its single envelope glycoprotein G which is both involved in receptor recognition and fusion of viral and cellular membranes. Here, we have characterized the fusion properties of CHAV-G. As for vesicular stomatitis virus (VSV, the prototype of the genus) G, fusion is triggered at low pH below 6.5. We have also analyzed the biochemical properties of a soluble form of CHAV-G ectodomain (CHAV-G_th_, generated by thermolysin limited-proteolysis of recombinant VSV particles in which the G gene was replaced by that of CHAV). The overall behavior of CHAV-G_th_ is similar to that previously reported for VSV-G_th_. Particularly, CHAV-G_th_ pre-fusion trimer is not stable in solution and low-pH-induced membrane association of CHAV-G_th_ is reversible. Furthermore, CHAV-G_th_ was crystallized in its low pH post-fusion conformation and its structure was determined at 3.6Å resolution. An overall comparison of this structure with the previously reported VSV-Gth post-fusion conformation, shows a high structural similarity as expected from the comparison of primary structure. Among the three domains of G, the pleckstrin homology domain (PHD) appears to be the most divergent and the largest differences are confined to the secondary structure of the major antigenic site of rhabdoviruses. Finally, local differences indicate that CHAV has evolved alternate structural solutions in hinge regions between PH and fusion domains but also distinct pH sensitive switches. Globally the comparison between the post fusion conformation of CHAV and VSV-G highlights several features essential for the protein’s function. It also reveals the remarkable plasticity of G in terms of local structures.

## Author Summary

Chandipura virus (CHAV, belonging to vesiculovirus genus), is an emerging pathogen associated with deadly encephalitis, principally among children, in India. CHAV single envelope glycoprotein G is involved in two successive steps of virus entry: receptor recognition that allows endocytosis of the virion and fusion of viral and cellular membranes that allows release of the viral nucleocapsid into the cytoplasm for the subsequent steps of infection. As for other rhabdovirus, fusion is triggered at low pH and catalyzed by a conformational change of G from its pre-fusion toward its post-fusion conformation. We have determined the crystalline structure of CHAV-G in its post-fusion conformation. An overall comparison of this structure with the previously reported post-fusion conformation of vesicular stomatitis virus G reveals the selective pressure of the humoral immune response: The major antigenic site, located in the most exposed domain of the pre-fusion conformation of the glycoprotein, is also the most structurally divergent region of G. The structure also highlights the remarkable plasticity of G in terms of local structures particularly in some hinge regions and reveals that the vesiculovirus can be classified in two groups based on the nature of their pH sensitive switches which trigger the conformational change of G.

## Introduction

Chandipura virus (CHAV; Family Rhabdoviridae, Genus *Vesiculovirus*) is an emerging human pathogen associated with deadly encephalitis, principally among children in the tropical areas of India. CHAV can be isolated from natural populations of *Phlebotomine* sandflies [1–3] and, in the recent years, has caused several outbreaks with high mortality rate [4]. Despite its importance in public health, there are no structural data available on CHAV proteins. In fact, most of the functional and structural information on CHAV has been inferred from studies performed on the prototype vesiculovirus, vesicular stomatitis virus serotype Indiana (VSV_IND_). This inference is reasonable due to the conservation of the amino acid (aa) sequences of the proteins of the two viruses [5]; for instance, aa sequences of glycoproteins G from both viruses share 40% identity and around 65% similarity [6].

The entry of rhabdoviruses into the host cell is mediated by glycoprotein G which constitutes the spikes that protrude at the viral surface [7]. First, G mediates viral attachment via interaction with a cellular receptor that, in the case of VSV_IND_, has been demonstrated to be the LDL receptor and its family members [8]. Then, after endocytosis [9,10], upon acidification of the inner environment of the endosome, G undergoes a low-pH-induced structural transition from its trimeric pre-fusion form (PRE) towards its trimeric post-fusion conformation (POST) that drives fusion between the viral and endosomal membranes [11,12]. During this conformational change, internal hydrophobic motifs (the so-called fusion loops) interact with one or both participating membranes [13], resulting in their destabilization and merger. Remarkably, low-pH-induced structural transition of rhabdoviral G is reversible [11,14]. In fact, there is a pH dependent thermodynamic equilibrium between different states of G. At neutral pH, G exists as a population of mostly monomeric species with a minority of PRE trimers [12,15] whereas, at low pH, the G distribution is shifted toward the trimeric POST state [15,16]. This is the main difference between rhabdoviral G and other viral fusion glycoproteins activated at low pH for which the PRE conformation is metastable [17,18].

Mature glycoprotein G is about 500 aa long (495 for VSV_IND_ and 509 for CHAV). G has a huge ectodomain (452 aa for CHAV-G) anchored in the lipid bilayer by a single transmembrane domain (TMD) which is located upstream a short C-terminal intra-viral segment. The crystal structures of both PRE and POST states of VSV_IND_-G ectodomain (VSV_IND_-G_th_, obtained by limited proteolysis of G at the surface of virions of the Indiana Mudd-Summers strain with thermolysin) have been previously reported [19,20]. This revealed that the POST structure of VSV-G has the same fold as the presumptive post-fusion state of glycoprotein gB of herpesviruses [21] and baculovirus gp64 [22]. This defined a new class (class III) of fusion proteins [23].

G ectodomain folds into three distinct structural domains [24] (S1 Fig.). A central domain (CD) is made of a lateral β-sheet rich region and a central helix which is involved in trimerization of the molecule in both PRE and POST states. A pleckstrin homology domain (PHD) is inserted in a loop of CD and the fusion domain (FD) is itself inserted in a loop of PHD. The FD is made of an extended β-sheet structure at the tip of which are located the two hydrophobic fusion loops. These rigid domains are connected by segments (R1–4) which, together with the C-terminal segment (R5) connecting CD to TMD, undergo major refolding events during the structural change.

During the conformational change from PRE to POST conformation, both FD and the C-terminal part of the ectodomain rotate around CD [20,24] (S1 Fig.). As a consequence, both the fusion loops and the TM domain move from one end of the molecule to the other. The FD moves relatively to PHD due to refolding of segments R2 and R3. There is also a major refolding event in the two segments connecting CD to PHD (R1 and R4) leading to lengthening of the CD central helix F. Similarly, the C-terminal segment of the ectodomain both completely relocates and refolds into α-helix H, which positions itself, in an antiparallel manner, in the grooves of the trimeric core made by the three helices F. This results in the formation of a six-helix bundle that buries acidic amino acids, which constitute pH sensitive molecular switches [19,25]. The complex conformational change has been demonstrated to involve monomeric intermediates [15].

In this work, we have characterized the fusion and biochemical properties of CHAV-G and compared them to those of VSV-G. We also determined the structure of a soluble form of CHAV-G ectodomain (CHAV-G_th_, generated by thermolysin limited-proteolysis of recombinant VSV particles in which the G gene was replaced by that of CHAV[26]) in the POST conformation at 3.6 Å resolution. An overall comparison of this structure with VSV-G_th_ POST, shows a high structural similarity as expected from the comparison of aa sequences. Among the three domains of G, the PHDs appear to be the most divergent and the largest differences are confined to the secondary structure of the major antigenic site of rhabdoviruses. Finally, local differences indicate that CHAV, together with a related group of vesiculoviruses, has evolved alternate structural solutions in hinge regions between PHD and FD and distinct pH sensitive switches.

## Results and Discussion

### Fusion properties of CHAV glycoprotein

We compared the fusion activity of both CHAV-G and VSV-G in a cell-cell fusion assay. For this, BSR cells were co-transfected with a plasmid encoding G (either VSV-G or CHAV-G) and rabies virus (RABV) phosphoprotein fused to the green fluorescent protein (P-GFP). P-GFP is unable to passively diffuse into the nucleus [27]. This allows an easy visualization and counting of multinucleated cells (syncytia) formed as a result of the fusion activity of the glycoprotein [25,28]. Transfected cells were incubated for 10 minutes with medium adjusted to pH values from 5 to 7, which was then replaced by medium buffered at pH 7.4. The cells were then further incubated for 1h at 37°C before fixation.

**Fig 1:**
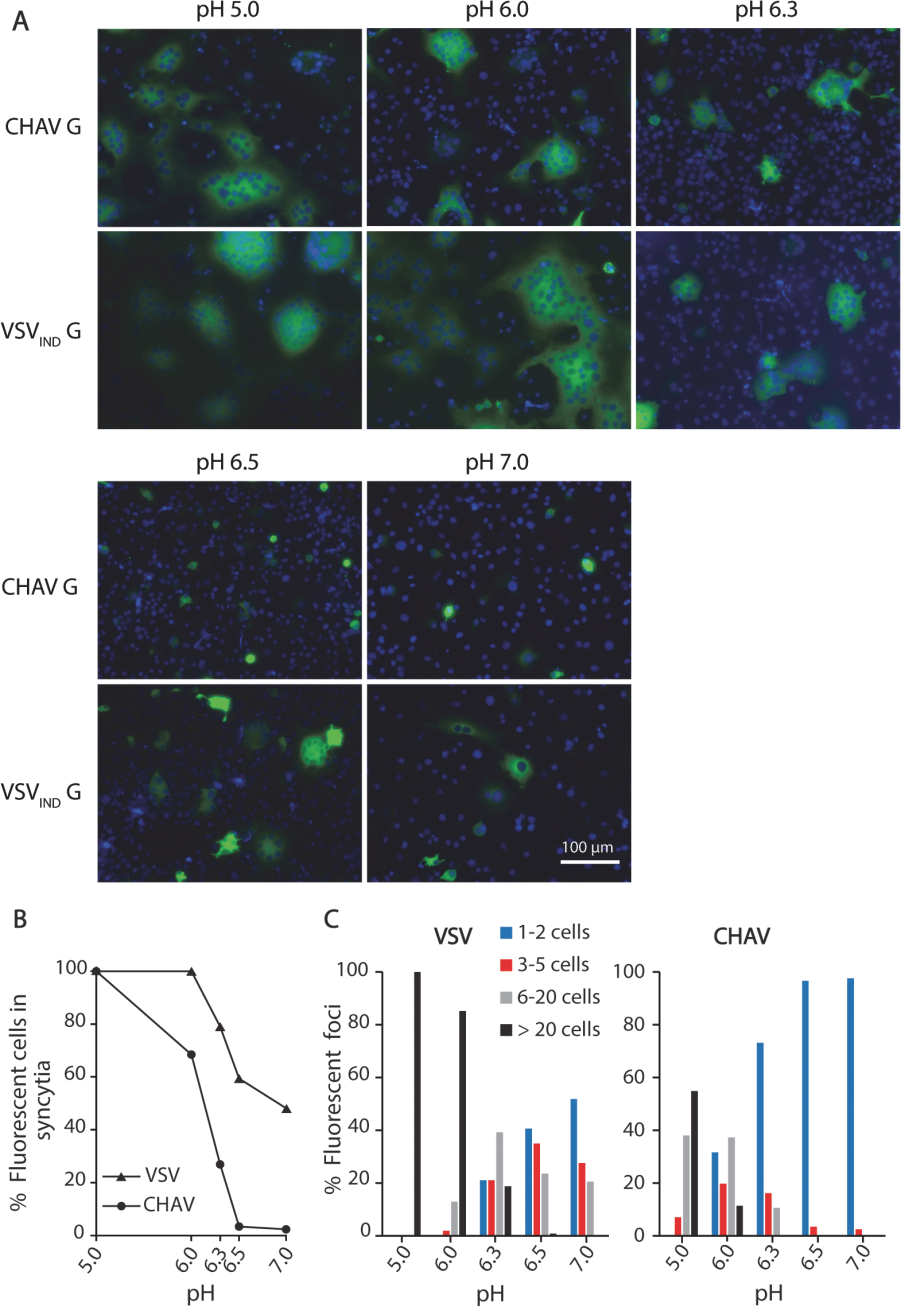
Fusion activity of CHAV-G and VSV-G analyzed in a cell-cell fusion assay. A) BSR cells were transfected with plasmids expressing the full-length G proteins and RABV P-GFP to facilitate the observation of syncytia. At 24 h post-transfection, the cells were incubated for 10 min with cell medium adjusted to the indicated pH, and then replaced by fresh medium at pH 7.4. The cells were kept at 37°C for 1 h before fixation. Nuclei were stained with DAPI. All images are representative examples from at least three independent experiments. The scale is the same for all micrographs. B) Percentage of fluorescent cells having formed a syncytia (containing more than 3 nuclei) as a function of pH for both VSV_IND_-G and CHAV-G. C) Distribution of the size of syncytia as a function of pH for both VSV_IND_-G and CHAV-G (at each pH, the examined field corresponded to about 400 transfected fluorescent cells at pH7).

We could observe formation of massive syncytia between pH 5.0 and 6.0 for cells expressing either CHAV-G or VSV_IND_-G. At pH 6.3, syncytia were smaller for both glycoproteins. At pH 6.5, no syncytia were detected for CHAV-G whereas small syncytia (less than 20 nuclei) were still observed for VSV_IND_-G up to pH 7.0 (Fig. 1).

### Purification and biochemical properties of CHAV-G_th_

We purified CHAV-G ectodomain from a recombinant VSV in which the G gene was replaced for that of CHAV (VSV/CHAV-G) [29]. This chimeric virus is non-pathogenic and can be produced at high titers in BSR cells. At pH 6.0, thermolysin produces a single cut in CHAV-G between residues 419 and 420 (the cleavage site was determined by mass spectrometry). After proteolysis, the CHAV-G ectodomain (CHAV-G_th_, residues 1–419) was purified by successive steps of ultracentrifugation and chromatography as previously described [26].

In the case of VSV-G, the pre-fusion trimer, although detected at the viral surface [12], is not stable in solution and only monomers of G are detected at high pH [11,15]. On the other hand, at low pH, the post-fusion trimer is stable [11,15]. Monomers and post-fusion trimers of G have distinct sedimentation coefficient and can be easily separated in a sucrose gradient [11,25]. Therefore, we analyzed the quaternary structure of CHAV-G_th_ on linear 5–20% (w/v) sucrose gradients at different pHs and compared it with that of VSV_IND_-G_th_.

**Fig 2:**
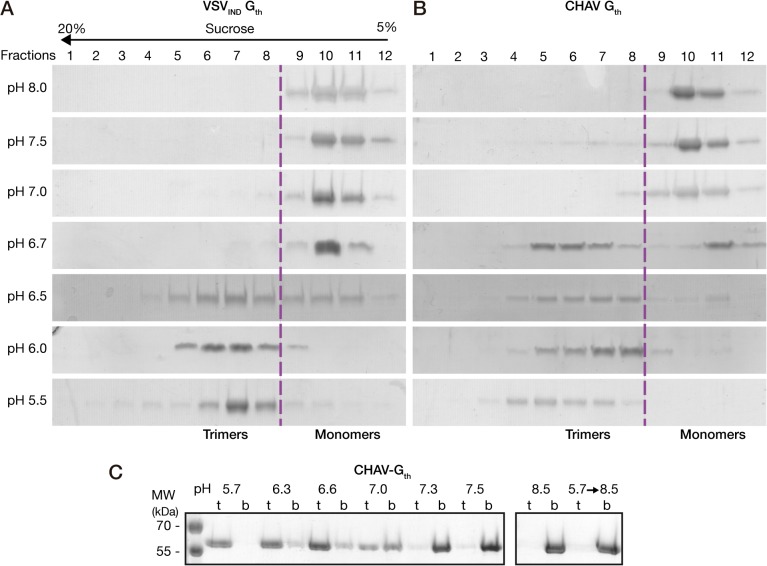
Biochemical properties of CHAV-G_th_ and VSV-G_th_. Oligomerization assay of CHAV-G_th_ (**A**) and VSV-G_th_ (**B**). Proteins were preincubated in 50 mM TRIS or MES buffers adjusted to the indicated pH. The solution was loaded on a linear 5–20% sucrose gradient at the same pH and spun at 35000 rpm for 16 h and 4°C. After centrifugation, 12 fractions were collected and analyzed by 10% SDS-PAGE gels stained with Coomassie blue. The dashed line indicates the gradient boundary between monomeric pre- and trimeric post-fusion forms. **C)** Membrane flotation assay of CHAV-G_th_ incubated with liposomes over a range of pH values from 5.7 to 8.5. For each experiment, top (t) and bottom (b) fractions were collected and analyzed on 10% SDS PAGE stained with Coomassie Blue. The right panel shows the experiments performed at pH 8.5 and the reversibility of the low-pH-induced association of G_th_ with liposomes after incubation at pH 5.7 followed by a re-incubation at pH 8.5.

The overall behavior of CHAV-G_th_ in the sucrose gradients was similar to that of VSV_IND_-G_th_ (Fig. 2A and 2B). At pH values from 7.0 to 8.0, G_th_ was found in fractions 9–12 corresponding to monomeric species, while at pH 5.5 and 6.0 only the trimeric post-fusion form was found in lower fractions of the gradient (fractions 4–8). However, the pK of the transition (defined as the pH value at which monomeric and trimeric G are found in equal amounts) was slightly higher for CHAV-G_th_ (pH 6.7) than for VSV-G_th_ (pH 6.5).

We also investigated the membrane interaction properties of CHAV-G_th_ by liposome flotation assay. CHAV-G_th_/liposome interaction was assessed over a broad pH-range, from pH 5.7 to pH 8.5 (Fig. 2C). From pH 5.7 to 6.6, all the glycoprotein was located in the upper layer indicating a massive association of CHAV-G_th_ with liposomes. At pH 7.0, the quantity of CHAV-G_th_ recovered in the bottom fraction increased considerably and only half of the glycoprotein was still detected in association with membranes. From pH 7.3 to 8.5, CHAV-G_th_ was exclusively found in the bottom fraction indicating a poor membrane interacting activity. Finally, when CHAV-G_th_-liposome complexes formed at pH 5.7 were brought to pH 8.5, membrane flotation experiments revealed a complete dissociation of glycoproteins from liposomes.

Taken together, our results indicate that the fusion properties and the associated low-pH-induced conformational change are very similar for CHAV-G and VSV-G. Particularly, as VSV-G [11,15], CHAV-G pre-fusion trimer is not stable in solution and low-pH-induced membrane association of CHAV-G is reversible.

### Structure of the post-fusion conformation of CHAV-G_th_

We screened several crystallization conditions for CHAV-G_th_ in presence of n-dodecyl β-maltoside (DDM) to avoid rosette formation via aggregation of the protein through its fusion loops. In these experiments, we could obtain CHAV-G_th_ crystals at pH 4.6 and determine the structure of the protein at 3.6 Å resolution by molecular replacement. Statistics of data collection and refinement are given in Table 1. The comparatively low resolution of the structure and starting phases obtained from molecular replacement with a 40% identity model (only 25% between PH domains) did not preclude an accurate final model in this case. This is due to a large solvent content and three molecules in the asymmetric unit. This yields over 20,000 unique reflections with which to refine three times 3,250 atoms per protomer with tight non-crystallographic symmetry and stereochemical restraints and conservative temperature factor models (see Material and Methods section for more details). The structure's accuracy is attested by excellent final statistics, particularly agreement between model and data and stereochemical parameters including the most unbiased indicators (Table 1, R-free and Ramachandran plot, respectively). Final temperature factors are quite reasonable for this resolution except in the distal parts of fusion domains, in accordance with these parts being the most flexible tip of an elongated molecule (S2 Fig.). The parts omitted in the initial molecular replacement model showed density and could be built *de novo* after the first few rounds of refinement and rebuilding, and the final composite simulated annealing omit map confirms unambiguous placing of main chain and most side chains except in the distal parts of FD (S3 Fig.). Accordingly, in the following sections, we discuss only those parts of the model, particularly the hydrogen bond networks, for which density is unambiguous.

**Table 1:** Data collection and refinement statistics.

Data Collection	
Wavelength (Å)	1.167
Resolution range (Å)	49.13–3.6 (3.764–3.6)
Space group	C 1 2 1
Cell Dimensions	
a, b, c (Å)	365.59 83.5 60.82
α, β, γ (°)	90 96.95 90
Total reflections	66563 (6713)
Unique reflections	21199 (2087)
Multiplicity	3.1 (3.2)
Completeness (%)	99.33 (99.62)
Mean I/sigma(I)	7.49 (1.79)
Wilson B-factor	86.32
R-merge	0.1399 (0.6315)
R-meas	0.1684
CC1/2	0.993 (0.724)
CC*	0.998 (0.917)
**Refinement**	
Reflections used for R-free	1056
R-work	0.1966 (0.2421)
R-free	0.2595 (0.3434)
Number of atoms	
non-hydrogen	9745
macromolecules	9661
ligands	80
water	4
Protein residues	1235
RMS(bonds)	0.010
RMS(angles)	1.31
Ramachandran plot (%)	
favored	97
allowed	2.51
outliers	0.49
Clashscore	11.04
Average B-factor	101.70
macromolecules	101.30
ligands	158.90
solvent	33.60

Statistics for the highest-resolution shell are shown in parentheses.

The single trimer in the asymmetric unit has the three protomers in a classic post-fusion conformation. The segments R1 to R5 connecting the different domains were traced in the experimental electron density map. The three chains were built from residues 1 to 415, 1 to 416 and 1 to 413, for chains A, B and C, respectively; with internal breaks in segments 26–33 and 27–33 of chain A and C. Those breaks reflect the intrinsic flexibility of segment R1.

**Fig 3:**
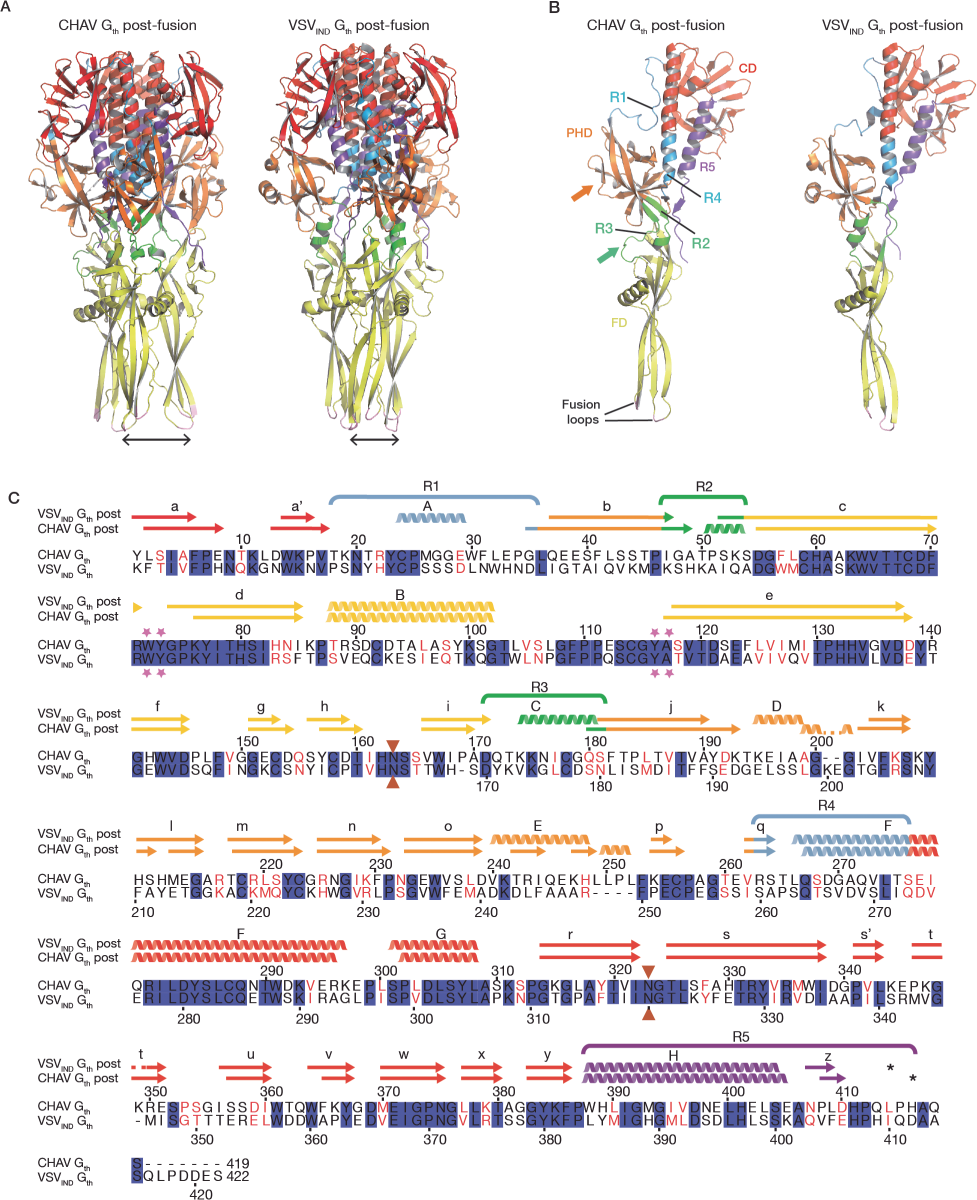
Overall structures of CHAV-G_th_ and VSV_IND_-G_th_ post-fusion forms. (**A**) Structure of CHAV-G_th_ and VSV_IND_-G_th_ post-fusion trimers and (**B**) their respective protomers. The proteins are colored by domains and segments as defined in Table 2. The fusion loops at the tip of FD are in pink. The arrows indicate the most noticeable differences between both proteins (located in PHDs and segments R3). The black double headed arrows indicate the spacing between the tips of FDs (between Cα of residue 73 and Cα of residue 117 of the neighboring protomer). The corresponding distance is 15.7Å for VSV- G and 26.2 Å for CHAV-G. **(C)** Sequence alignment of CHAV-G and VSV_IND_-G. Conserved residues are in blue boxes whereas similar ones are in red (overall aa sequence conservation is 41%). The black dashes show gaps. Elements of secondary structure are indicated above the sequence for the two structures. They are colored as the domain they belong to, with the color code of Table 1. VSVIND-G_th_ helices and sheets are named as in [20]. Segments R1 to R5 that refold during the transition are also indicated with the color code of Table 1. Brown triangles indicate the glycosylation sites. Residues located in the fusion loops are labeled by pink stars. The asterisks indicate the last residue of the molecule for which electron density was still interpretable.

The overall structure of CHAV-G_th_ is very similar to that of VSV_IND_-G_th_ in its post-fusion form (Fig. 3A and B) [19]. The structural comparison between CHAV-G and VSV_IND_-G reveals that the three domains are more conserved than would be expected from the sequence divergence [30,31] (Table 2, Fig. 3C). CDs and the upper part of FDs (excluding the poorly defined extremity of FD) main chains are almost superimposable within experimental error (Table 2, S4 Fig.).

**Table 2:** Domains and segments nomenclature used in the text.

Part of the molecule	Color	VSV_IND_ residues	CHAV residues	sequence identity	RMSD	
					VSV_IND_ PRE / VSV_IND_ POST	VSV_IND_ POST / CHAV POST
CD	Red	1–17 and 273–382	1–17 and 277–386	51%	0.68 Å (126 Cα)	1.2 Å (126 Cα)
PHD	Orange	36–46 and 181–259	36–45 and 183–262	25%	0.63 Å (90 Cα)	1.68 Å (79 Cα)
PHD core	-	36–45, 182–192, 196–199, 201–239, 241–244, 246–249, 250–259	36–45, 183–193, 196–199, 200–238, 239–242, 248–251, 253–262	25%	-	-
FD	Yellow	53–172	55–170	55%	-	-
FD upper part	-	55–61, 85–105, 127–170	55–61, 85–105, 127–170	43%	-	0.77 (65 Cα)
PHD core+ FD upper part	-	-	-	34%	-	1.57 (150 Cα)
R1	Cyan	18–35	18–35	-		
R2	Green	47–54	46–54	-		
R3	Green	173–180	171–182	-			
R4	Cyan	260–272	263–276	-		
R5	Purple	383–413	387–410	-		

Root mean square deviation (RMSD) and number of superimposable alpha carbons (Cα) are reported between indicated domains. As the distal (fusion loop-containing) part of FD is the most flexible part in all structures, we defined a “FD upper part” which was used to calculate the RMSD between VSV and CHAV-G_th_. We also defined a PHD core corresponding to the 79 residues (out of 90) in CHAV-G which are in structurally equivalent positions in VSV-G. The RMSD between the two (PHD core + FD upper part) is not significantly different from the value obtained for the PHD core alone. This indicates that the relative orientation of the two domains is the same in both proteins.

### PHD is the most divergent domain of vesiculovirus glycoproteins

**Fig 4:**
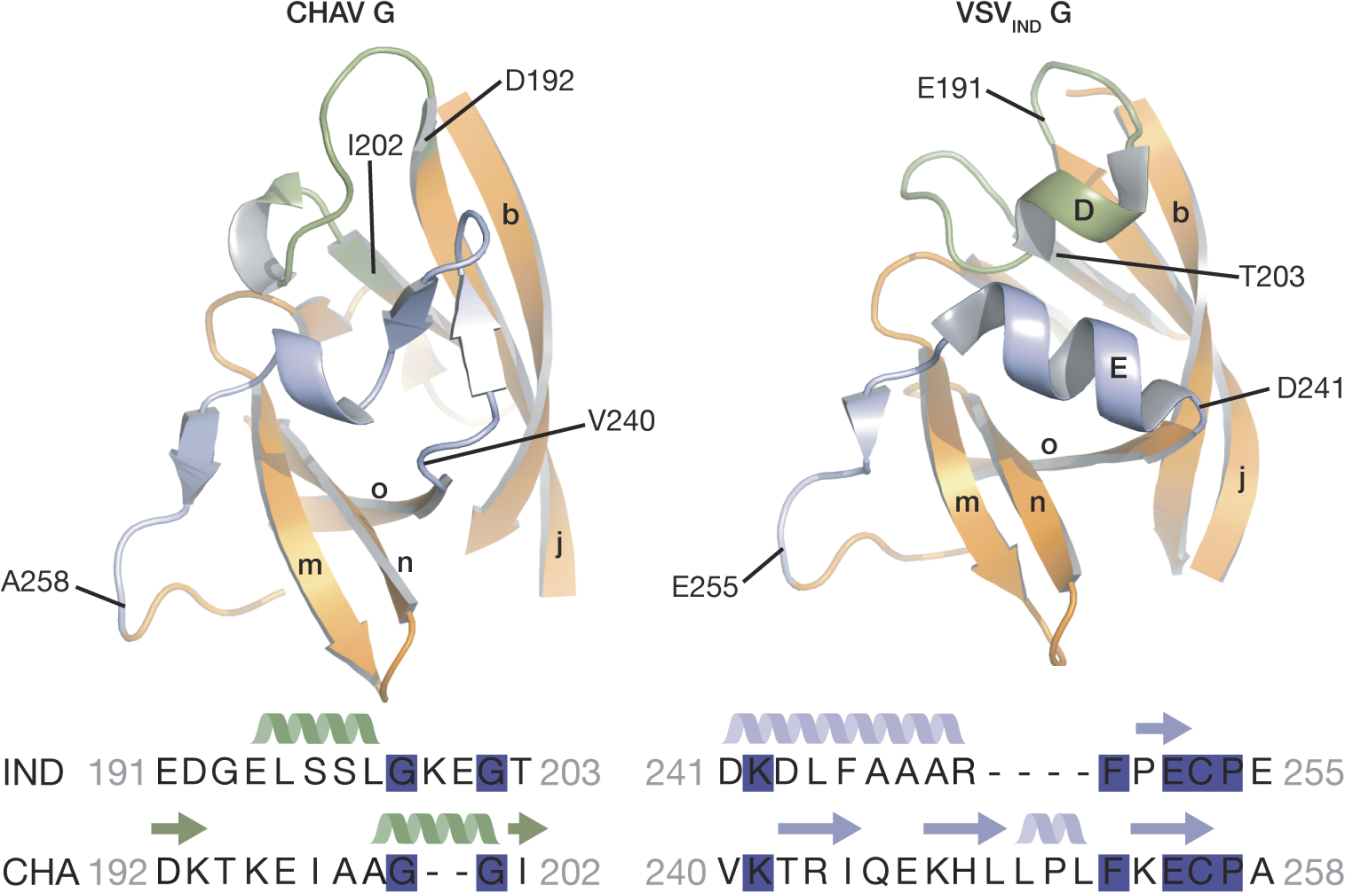
Ribbon representation of CHAV-G and VSV-G PHDs. The segments having different structures between the two domains and corresponding to Rhabdoviruses major antigenic site are highlighted in green and purple. Helices and β-sheets are labeled in capital and lower case letters, respectively, according to the previous VSV-G nomenclature. The sequence alignment below the structures corresponds to the two segments highlighted in the ribbon representation. The position of the first and last aa residues of the two sequences is indicated on the structures. Conserved residues between CHAV-G and VSV-G are highlighted in blue.

The PHDs are more divergent than FDs and CDs. Only 79 residues of the PHD (called PHD core) out of 90 in CHAV-G are in structurally equivalent positions with VSV-G (Table 2). The largest differences are confined to helices D and E which are missing in CHAV-G (see the orange arrow on Fig. 3B). A comparison of the sequence of both proteins at these positions reveals that CHAV-G bears an insertion of 4 aa residues at the level of helix E resulting instead in the formation of a small two stranded β-sheet (Fig. 4). Also, in the equivalent position to helix D, CHAV-G has a deletion of two residues leading to a loop conformation (Fig. 4).

VSV-G segments containing both helix D and helix E are the most exposed part of the glycoprotein in its PRE conformation at the viral surface and, in fact, this region constitutes the major antigenic site of several rhabdovirus glycoproteins [32–35]. This suggests that the structural divergence between CHAV-G and VSV_IND_-G in this region is the result of the selective pressure from the humoral response of the host immune system.

### Distinct structural solutions in the hinge region connecting PHD and FD

A second distinct feature between CHAV-G and VSV_IND_-G lies in the hinge R2 and R3 connecting both PHD and FD (green arrow on Fig. 3B). Although the relative orientation of the two domains is the same in both proteins (Table 2), there are differences in the secondary structure of these hinges.

**Fig 5:**
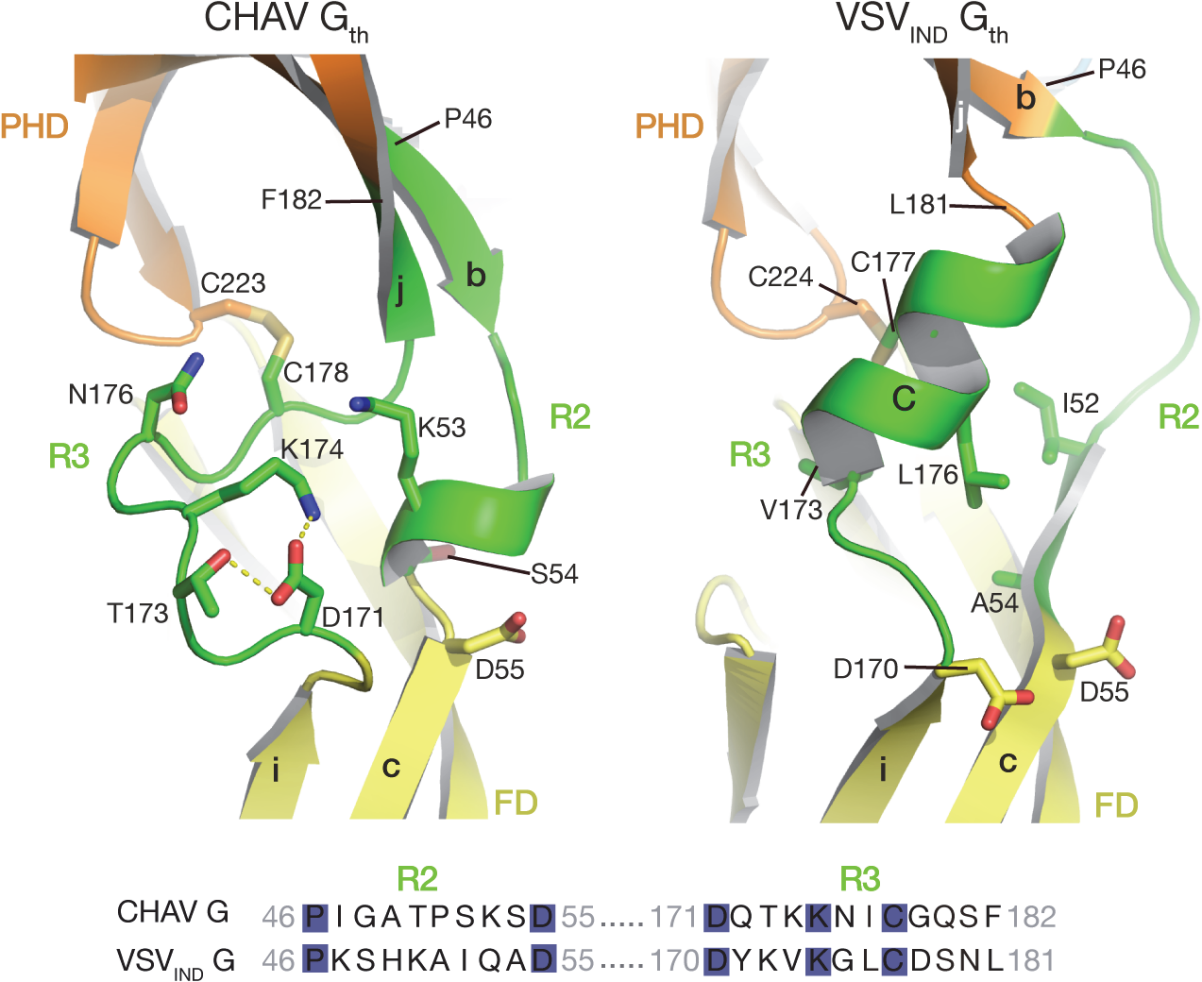
Ribbon representation of CHAV-G and VSV_IND_-G R2-R3 hinge regions. Protein domains are colored according to Table 2. The main elements of the secondary structure of VSV_IND_-G and their equivalents in CHAV-G are indicated with capital (for α-helices) and lower case (for β-strands) letters. Residues located at the interface between R2, R3 are in sticks representations. Note their polar character in the case of CHAV-G. The sequence alignment of segments R2 and R3 is displayed below the structures. The position of the first and last aa residues of the two sequences is indicated on the structures. Conserved residues between CHAV-G and VSV-G are highlighted in blue.

In the POST conformation of VSV_IND_-G, R2 is mainly a large loop and extends until the first residues of the β-strand c of FD, whereas R3 forms helix C. The contact between the two segments essentially involves hydrophobic residues (Fig. 5).

For CHAV-G, the amino-terminal part of R2 adopts a β-strand conformation, which is followed by a small loop and a small helix; CHAV-G R3 has a structure which is similar to VSV-G R2, a long loop ending in a β-strand. The interaction between the two segments is mediated by charged and polar residues (Fig. 5).

Despite those differences in R2 and R3 regions, the relative positions of FD and PHD are identical for both glycoproteins (Table 2). This suggests that the hinge regions need only to be flexible to allow the rotation of the FD.

Alignment of G aa sequences for 12 vesiculoviruses (S5 Fig.) reveals that the sequences of R2 and R3 segments are rather divergent among vesiculoviruses. This suggests that the structural constraints (*i*.*e*. flexibility and relative position of PHD and FD in the POST conformation) can be accommodated by very diverse amino acid sequences.

### Different pH sensitive molecular switches

**Fig 6:**
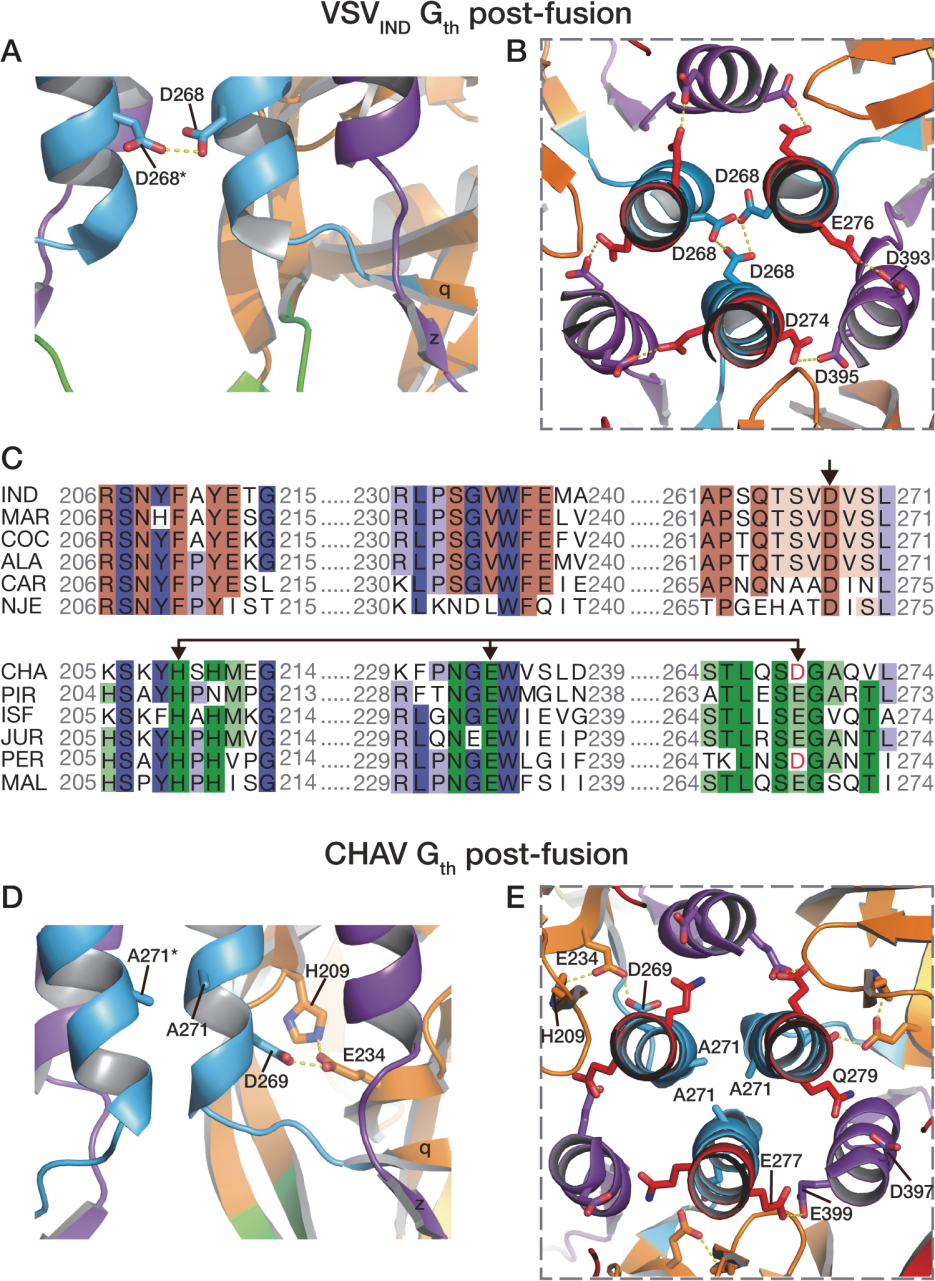
pH sensitive molecular switches in the post-fusion structures of VSV-G_th_ and CHAV-G_th_. pH sensitive molecular switches located in the 6-helix bundle of the post-fusion form of VSV_IND_-G (**A-B**) and CHAV-G (**D-E**). **A** and **D** are lateral views of two protomers interacting by the central helices F. **B** and **E** are top views of the trimeric interfaces for both proteins. The acidic character of VSV-G residues D274, D393 and D395 is conserved in CHAV-G whereas D268, a major pH sensor in VSV-G [25], is replaced by an alanine. Instead, in CHAV-G, another pH sensitive switch is formed by the triad H209, E234 and D269. **C:** Sequence alignments of 12 vesiculoviruses in regions flanking the molecular switches D268 (single arrow) and D269/D234 (double headed arrow) from VSV_IND_-G and CHAV-G, respectively. Vesicular Stomatitis Indiana Virus (**IND**, GenBank: AAA48370.1), Maraba Virus (**MAR,** GenBank: AEI52254.1), Cocal Virus (**COC,** GenBank: ACB47437.1), Vesicular Stomatitis Alagoas Virus (**ALA,** GenBank: ACB47442.1), Carajas Virus (**CAR,** Patent: JP 2010503660-A 2 04-FEB-2010), Vesicular Stomatitis New Jersey Virus (**NJE,** NCBI: YP_009047084.1), Chandipura Virus (**CHA,** NCBI: YP_007641380.1), Piry Virus (**PIR,** Swiss-Prot: Q85213.1), Isfahan Virus (**ISF,** NCBI: YP_007641385.1), Jurona Virus (**JUR,** GenBank: AEG25348.1), Perinet Virus (**PER,** GenBank: AEG25354.1), Malpais Spring Virus (**MAL,** GenBank: AGI04017.1). Conserved residues through the whole genus are in shade of blue, through the CHAV group are in shade of green and through the IND groups are in shades of brown.

Four acidic residues of VSV_IND_-G (D268, D274, D393 and D395), which are brought close together in the POST six-helix bundle (Fig. 6A and 6B), have been demonstrated to play the role of pH-sensitive switches for the transition back from the POST toward the PRE conformation of VSV-G [25]. The acidic character of the residues corresponding to D274, D393 and D395 of VSV-G is conserved among the vesiculovirus genus (S5 Fig.). However, this is not the case for the residue corresponding to D268 which is a major pH-sensitive switch of VSV_IND_-G [25].

Specifically, in CHAV-G, the residue at this position is an alanine (A271). The absence of this pH-sensitive switch seems to be compensated by CHAV-G’s residues D269 and E234 which form an intra-protomer hydrogen bond with H209 in their vicinity (Fig. 6D and 6E). This clustering is absent for the corresponding, and non-protonable, residues of VSV_IND_-G POST. Clustering of protonable residues is known to affect the pK_a_ of their side chain. Indeed, when the PropKa program [36] was used to compute the pKa of the side chains of the cluster based on CHAV-G POST structure, it predicted a pKa of about 10.5 for the carboxylate of residue D269. The only other residue with such a pKa shift in CHAV-G POST is residue E399 which corresponds to VSV_IND_-G D395 which has also been demonstrated to contribute to the pH sensitivity of VSV_IND_-G POST. Similarly, VSV_IND_-G D268 was calculated to have a pKa of about 14.5 in agreement with its established role as a major pH sensitive switch in VSV_IND_-G POST. This further suggests the likely role of the CHAV-G POST cluster D269, E234 and H209 as a pH sensitive switch. The deprotonation of these residues at high pH is certainly destabilizing the POST conformation of CHAV-G.

Remarkably, alignment of vesiculoviruses G aa sequences (Fig. 6) reveals that the vesiculoviruses can be classified in two groups. Either they have an aspartate residue in the position corresponding to VSV-G D268, or they have a histidine in position corresponding to CHAV-G H209 and an acidic residue in position corresponding to CHAV-G E234 and D269 (Figs. 6C and S5). Unsurprisingly, this segregation which relies only on vesiculovirus glycoprotein local sequences is in agreement with global phylogenetic studies on the genus [37,38].

**Fig 7:**
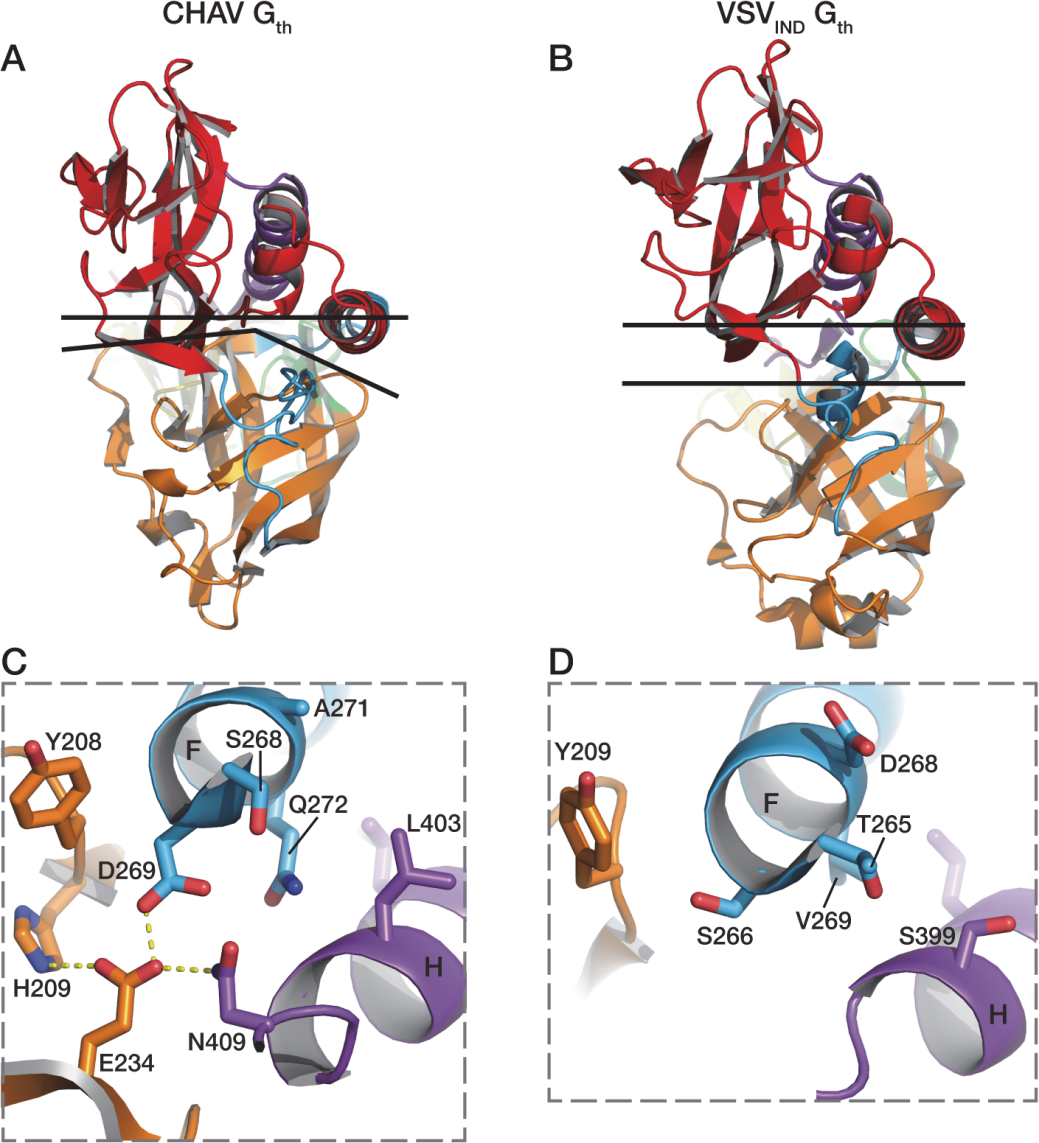
Packing of PHD domain against the central domain in CHAV-G_th_ and VSV_IND_-G_th_. In A and B are represented the top views of the protomers. The upper black lines are crossing the structures at the level of the Cα of two pairs of structurally equivalent residues in both glycoproteins which are S285 (located in helix F) and T10 for CHAV-G, and T282 and Q10 for VSV-G. The lower black lines show the border between PHD (orange) and CD (red). In C is shown the interactions at the base of helices F and H of CHAV-G_th_ involved in the close packing of PHD relative to CD. Residues implicated in the interaction and their immediate neighbors are in stick representation. In D are represented the equivalent residues of VSV_IND_-G.

This different switch of CHAV results in a tighter interaction between the base of the six-helix bundle and the PH domain (Fig. 7). As a consequence, the PHD has not exactly the same orientation relative to CD. This is why the FDs are slightly spread apart in CHAV-G (Fig. 3B).

### Final conclusions

The comparison of VSV_IND_-G and CHAV-G structures reveal that the domains do not evolve at the same rate and that the major actor on G evolution is the immune system. As a consequence, the most exposed domain in the PRE conformation (i.e. the PHD), which is the target of neutralizing antibodies, is also by far the most divergent domain among vesiculoviruses. This domain, located at the top of the PRE conformation, is also probably involved in receptor recognition. Therefore, it is probable that the humoral immune response is at the origin of receptor diversification among the rhabdovirus family.

The principal finding of this work is that, vesiculovirus G have diverged quite far in some respects but retained important functional and structural features. These features can be thus pointed at as essential for the protein’s function. As an example, the relative orientation of CD and PHD is different in CHAV-G and VSV-G POST protomers, but the relative orientation of PHD and FD remains the same despite the sequence and local structure divergence of segments R2 and R3 and the largely different sets of interactions between FD. This highlights the mechanistic importance of maintaining PHD-FD orientation. This also shows that the most important feature for segments R2 and R3 is their flexibility, but not their secondary structure. As a consequence, the structural constraints which exert on these segments are weak and R2 and R3 can tolerate quite different aa sequences. As another example, although pH sensitive switches are absolutely required for the correct function of G, those switches can be located in alternate places, as in the CHAV-G D269-E234-H209 cluster, the counterpart of which does not exist in VSV-G (Figs. 6C and S5).

In conclusion, the vesiculovirus glycoprotein, despite experiencing strong functional constraints, exhibits a remarkable plasticity in terms of sequence and even local structures. This is reminiscent of the remarkable structural conservation of the class II architecture despite the lack of any sequence similarity [39,40]. This suggests that evolvability is a key feature for viral fusion glycoproteins: They display a 3D structure that is robust in the face of amino acid sequence variation because of being malleable in local conformations, yet still maintaining the overall trimeric post-fusion conformation that is essential for their function.

## Materials and Methods

### Production and purification of CHAV-G_th_

CHAV-G_th_ was purified as previously described [26]. Briefly, a recombinant VSV harboring the CHAV-G gene [29] was produced at high titers in BSR cells, clones of BHK-21 (baby hamster kidney, ATCC CCL-10). Purified virions were submitted to proteolysis with thermolysin at pH 6.0 for 1.5h. This treatment generates a soluble fragment of G comprising the residues 1–419 (G_th_) as assessed by mass spectrometry. After digestion, the virus was pelleted and the viral pellet was suspended in 50 mM HEPES pH 7.5 containing 2% glycerol. The suspension containing G_th_ was clarified by ultracentrifugation at 25000 rpm (SW35 rotor) for 1h on a 20% w/v sucrose cushion. G_th_ purification was achieved on a DEAE trisacryl column (GE healthcare), followed by a size exclusion step using a Superdex S200 10/30 column (GE healthcare). Fractions containing G_th_ were concentrated up to 10 mg/ml and stored at −80°C until use.

### Cell-cell fusion assay

BSR cells plated on glass coverslips at 70% confluence were co-transfected with pCAGGS plasmids encoding VSV-G or CHAV-G, and P-GFP plasmid encoding the phosphoprotein of rabies virus fused to GFP [27]. Twenty four hours after transfection, cells were incubated with fusion buffer (DMEM-10mM MES or HEPES) at various pHs (from 5.0 to 7.0) for 10 minutes at 37°C. Cells were then washed once and incubated with DMEM-10mM HEPES-NaOH buffered at pH 7.4, 0.1% BSA at 37°C for 1 hour. Cells were fixed with 4% paraformaldehyde in 1× PBS for 15 min. Cells nuclei were stained with DAPI and syncytia formation was analyzed with a Zeiss Axiovert 200 fluorescence microscope at 20x magnification.

### Oligomerization assay

The oligomeric state of the G_th_ protein was determined by sucrose gradient centrifugation. 100 μg of VSV_IND_-G_th_ or CHAV-G_th_ were incubated in 200 μl of buffer containing either 50 mM MES-NaOH (for pH 5.5 to 6.7) or 50 mM Tris-HCl (for pH 7 to 8.5) plus 100 mM NaCl at 37°C for 30 minutes. The protein solution was overlaid on a 5-to-20% w/v continuous sucrose gradient in 100 mM NaCl, 50 mM Tris-HCl or 50mM MES-NaOH at the required pHs. Following centrifugation at 35,000 rpm for 16 hours in an SW41 rotor, 12 equal fractions were collected manually from the bottom of the gradient. When required, the fractions were dissolved and reconcentrated to the original volume using ultrafiltration devices (30 kDa molecular weight cut off) to get rid of the excess of sucrose. 20 μl of each fraction was analyzed on 10% SDS-PAGE gels stained with Coomassie blue.

### Membrane flotation assay

Membrane flotation assays were performed as previously described [15]. Briefly, 500 μg of fresh liposomes (phosphatidylcholine: phosphatidylethanolamine: gangliosides, 7:3:1) were incubated with 100 μg of purified G_th_ at the desired pH during 30 minutes at 37°C (final volume 600 μL). The lipid-protein mix was mixed with a 80% w/v sucrose solution so that final concentration of sucrose was around 65%. The mixture was overlaid with 2 mL of 50% w/v sucrose solution and 1.5 mL of 10% w/v sucrose solution, both solutions at the desired pH. Cushions were spun for 16 hr at 20°C in a SW55 Beckman rotor at 45,000 r.p.m. 20 μL of the top and bottom fraction were analyzed by SDS-PAGE. G_th_ in the top fraction was considered as liposome-bond, G_th_ in the bottom fraction was considered as not interacting with the membranes. For the reversibility experiment, lipid-protein mix was re-incubated at pH 8.5 after a first stage at pH 5.7.

### Crystallization of CHAV-G_th_ and structure determination

Crystals were obtained at 293 K by the hanging drop vapour diffusion method. Drops were prepared by mixing 1 μl of CHAV-G_th_ (4 mg/ml) supplemented with 0.2% DDM with 1 μl of the reservoir solution (12% PEG 3350, 0.1 M sodium acetate pH 4.6) and equilibrated against 500 μl of reservoir solution.

Crystals were harvested in a cryoprotecting solution containing reservoir solution supplemented with 30% glycerol and flash-cooled by plunging in liquid nitrogen. Diffraction data were collected at 100 K at PROXIMA 1 and ID-29 beam lines in SOLEIL and ESRF synchrotrons (France). Data for the best crystal were integrated and scaled using XDS/XSCALE [41]. The crystals belong to space group C2 with cell parameters consistent with 3 molecules in the asymmetric unit and 60% solvent content [42].

The structure was solved by molecular replacement (MR) with the PHENIX package [43]. The VSV-G post-fusion protomer (PDB 2CMZ, chain A) was first trimmed of non-conserved sidechains using the default procedure in phenix.sculptor. The three search models were the CD, FD and PHD further trimmed of carbohydrates and of a couple of residues upstream and downstream of refolding segments R1 to R5. The nine expected domains (corresponding to 3 protomers) in the asymmetric unit were placed by phenix.phaser. These initial phases were improved by solvent flattening and NCS averaging. Initial maps were clear enough to rebuild CDs and remove those parts of PHDs that did not fit the electron density maps. The model was iteratively rebuiltin COOT [44] and refined with phenix.refine. The refinement protocol was tailored to reduce the parameter to data ratio as much as possible: Tight non-crystallographic symmetry restraints were enforced between the three protomers in the asymmetric unit. B-factors were parameterized initially by TLS domains, allowing subsequent individual B-factor refinement with the very tight restraints on neighboring atoms implemented in recent versions of phenix.refine. This refinement protocol may allow very high B-factors in distal parts of elongated molecules due to the TLS component. The CDs, PHDs and the upper parts of FDs were carefully rebuilt and segments R1 to R5 added in as density became clear enough to model them. No Ramachandran restraints were applied in refinement at any stage to allow orthogonal validation of stereochemistry. All the structure figures were prepared using the program PyMol (PyMOL Molecular Graphics System. DeLano Scientific LLC, San Carlos, CA, USA. http://www.pymol.org).

## Supporting Information

S1 Fig Ribbon diagrams of the VSV_IND_-G_th_ pre-fusion and post-fusion protomer. G is depicted by domains colored as indicated in Table 2. The protomers are aligned on the CD (in red). The purple and yellow arrows respectively indicate the movement of the C-terminal R5 segment and the FD relative to the CD during the conformational change.
(TIF)

S2 Fig Ribbon diagram of the CHAV-G_th_ POST conformation colored according to the temperature factor. The regions of lowest B factors are colored in blue.
(TIF)

S3 Fig Selected regions of CHAV-G_th_ post-fusion model and accompanying electron density maps. The molecular switch H209/E234/D269 and neighboring amino acids (A, B), the R2-R3 hinge region (C, D) and the interface between helix H and F (E, F) are represented as sticks. Protein domains and segments are colored as described in Table 1. Key residues discussed in the text are indicated. The final refined 2F_OBS_-F_CALC_ map (A, C, E) and simulated-annealing composite omit map (B, D, F) are both contoured at 1σ. Omit map was calculated using phenix.refine [45].
(TIF)

S4 Fig Ribbon representation of the central domain (A) and the FD upper part (B) of VSV_IND_-G and CHAV-G. (TIF)

S5 Fig Sequence alignment of glycoproteins G from different vesiculovirus. Conserved residues are highlighted in shades of blue. The black dashes show gaps. The colored bars above the sequences indicate the domains described for CHAV-G and VSV_IND_-G. Residues located in the fusion loops are indicated by pink stars below the sequences. Residues involved in the formation of the pH sensitive molecular switches are indicated by dots below the sequences (in red are the residues making the switch which is specific of CHAV-G, in cyan is the residue making the switch which is specific of VSV-G, in purple are the residues contributing to pH sensing and common to both glycoproteins [25]). Vesicular Stomatitis Indiana Virus (IND), Maraba Virus (MAR), Cocal Virus (COC), Vesicular Stomatitis Alagoas Virus (ALA), Carajas Virus (CAR), Vesicular Stomatitis New Jersey Virus (NJE), Chandipura Virus (CHA), Piry Virus (PIR), Isfahan Virus (ISF), Jurona Virus (JUR), Perinet Virus (PER), Malpais Spring Virus (MAL). The accession number for all these sequences is given in Fig. 6.
(TIF)
