# Supplementary figures and images for: Structure of the Low pH Conformation of Chandipura Virus G Reveals Important Features in the Evolution of the Vesiculovirus Glycoprotein
Source: PLoS Pathog. 2015 Mar 24;11(3):e1004756. doi: 10.1371/journal.ppat.1004756 (PMC4372607; doi:10.1371/journal.ppat.1004756)

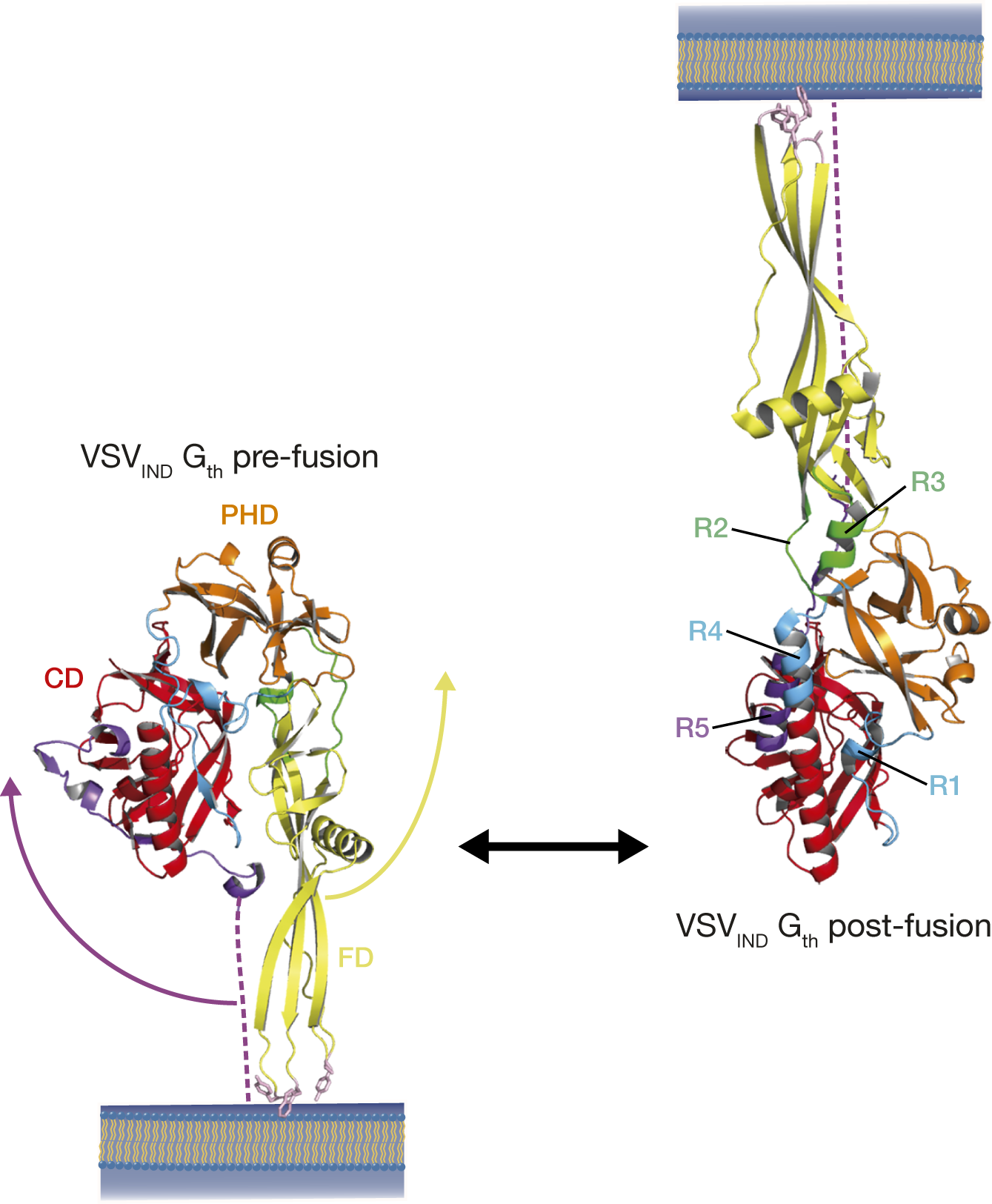

Supplement: S1 Fig — G is depicted by domains colored as indicated in Table 2. The protomers are aligned on the CD (in red). The purple and yellow arrows respectively indicate the movement of the C-terminal R5 segment and the FD relative to the CD during the conformational change. (TIF) [file ppat.1004756.s001.tif]

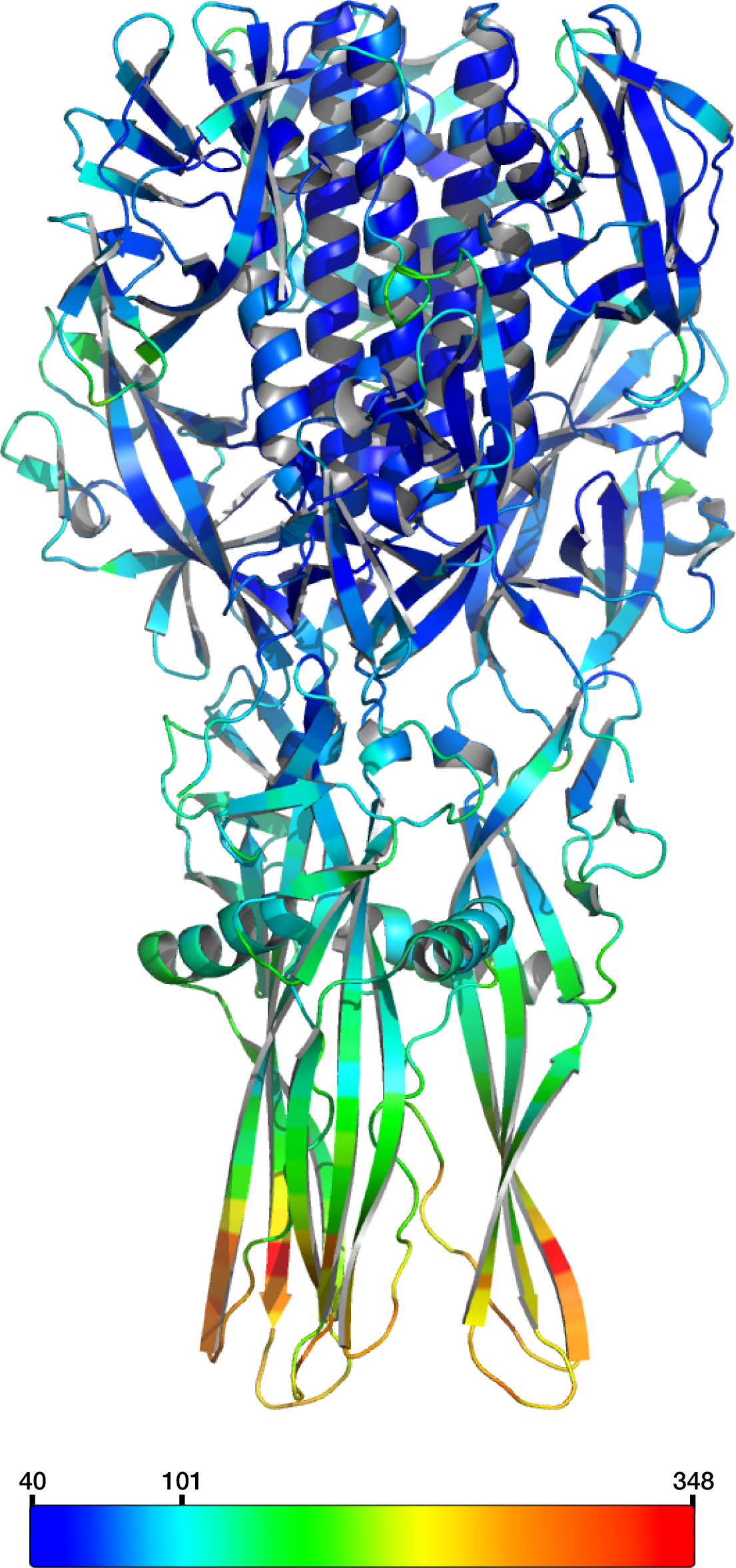

Supplement: S2 Fig — The regions of lowest B factors are colored in blue. (TIF) [file ppat.1004756.s002.tif]

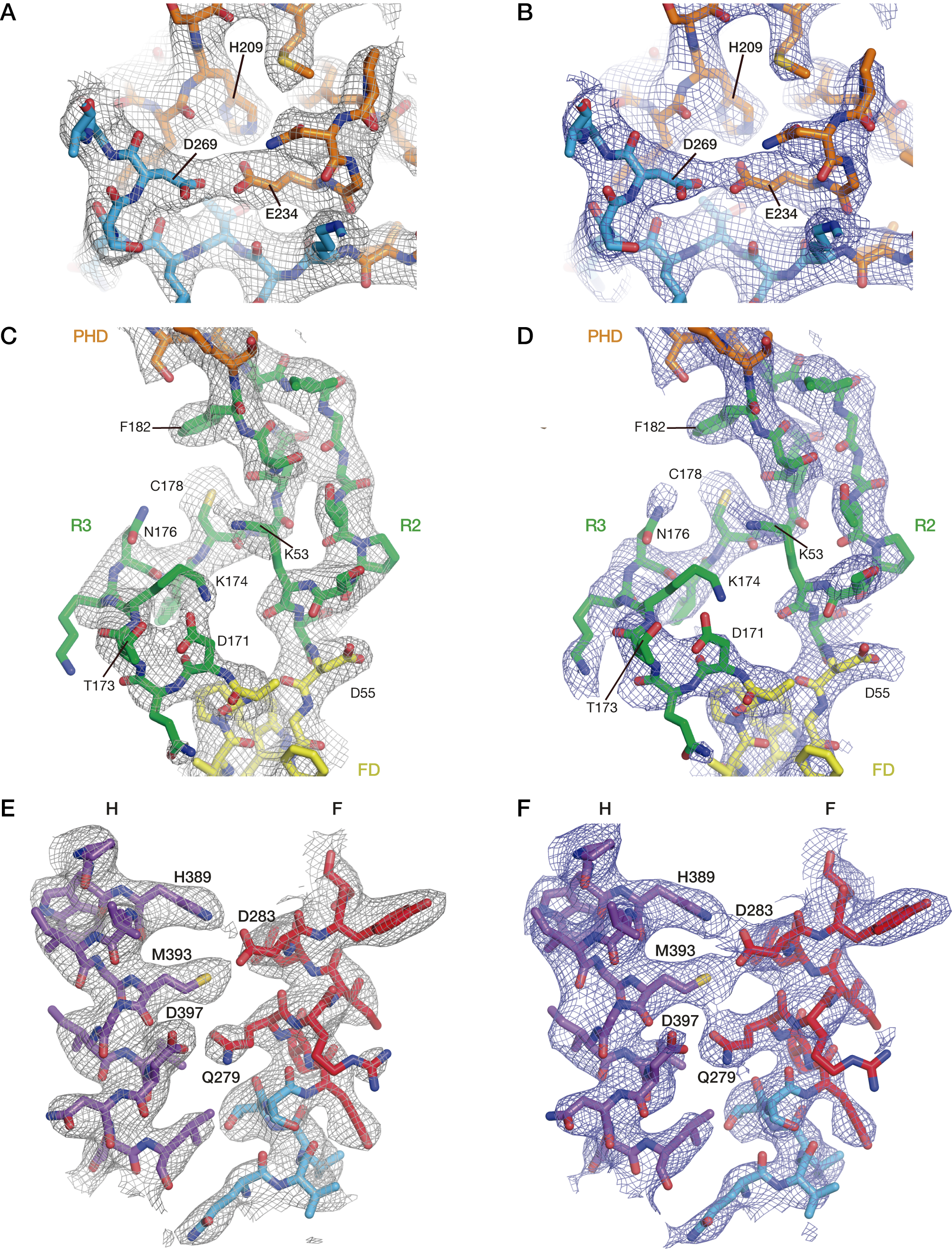

Supplement: S3 Fig — The molecular switch H209/E234/D269 and neighboring amino acids (A, B), the R2-R3 hinge region (C, D) and the interface between helix H and F (E, F) are represented as sticks. Protein domains and segments are colored as described in Table 1. Key residues discussed in the text are indicated. The final refined 2FOBS-FCALC map (A, C, E) and simulated-annealing composite omit map (B, D, F) are both contoured at 1σ. Omit map was calculated using phenix.refine [45]. (TIF) [file ppat.1004756.s003.tif]

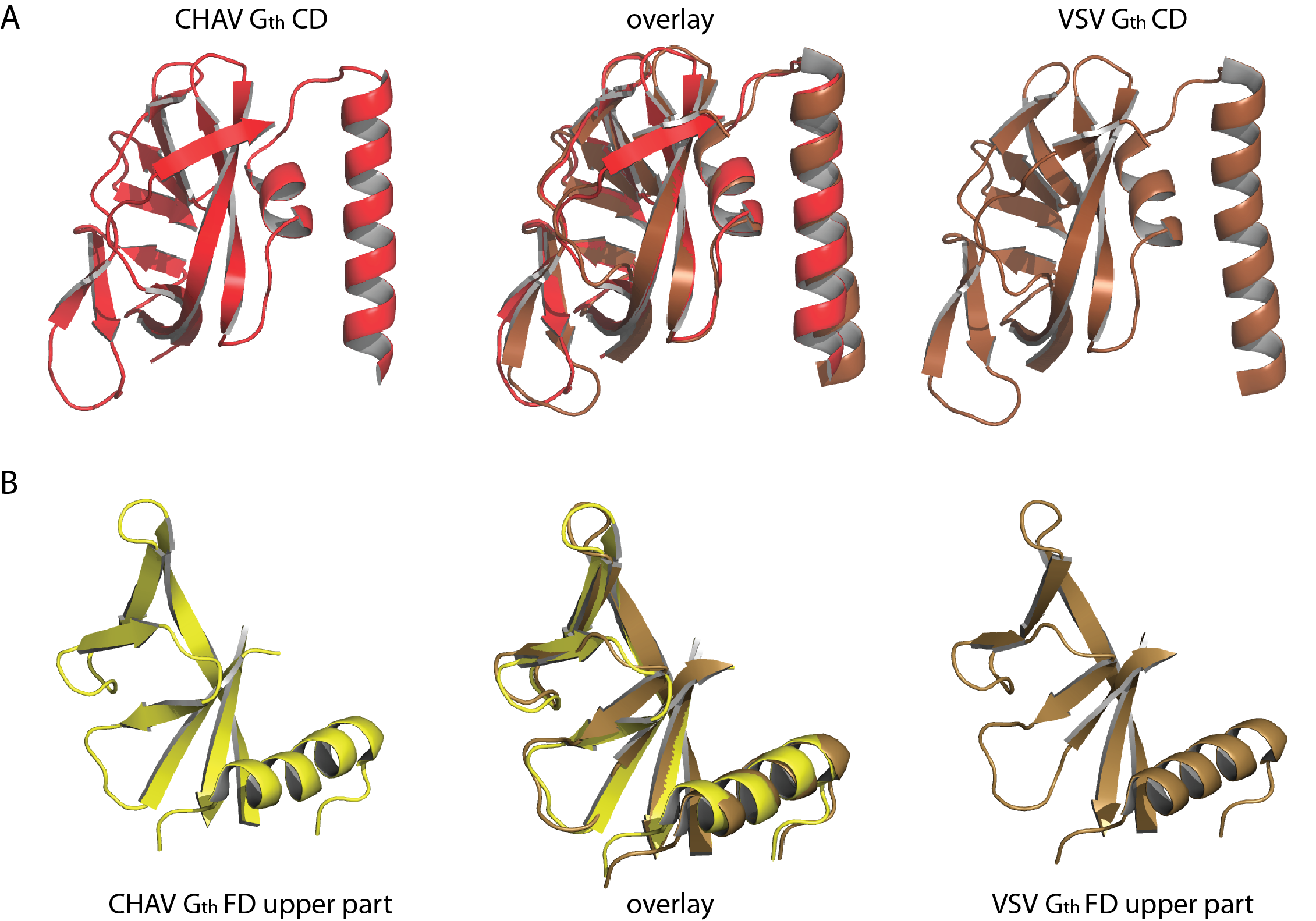

Supplement: S4 Fig — (TIF) [file ppat.1004756.s004.tif]

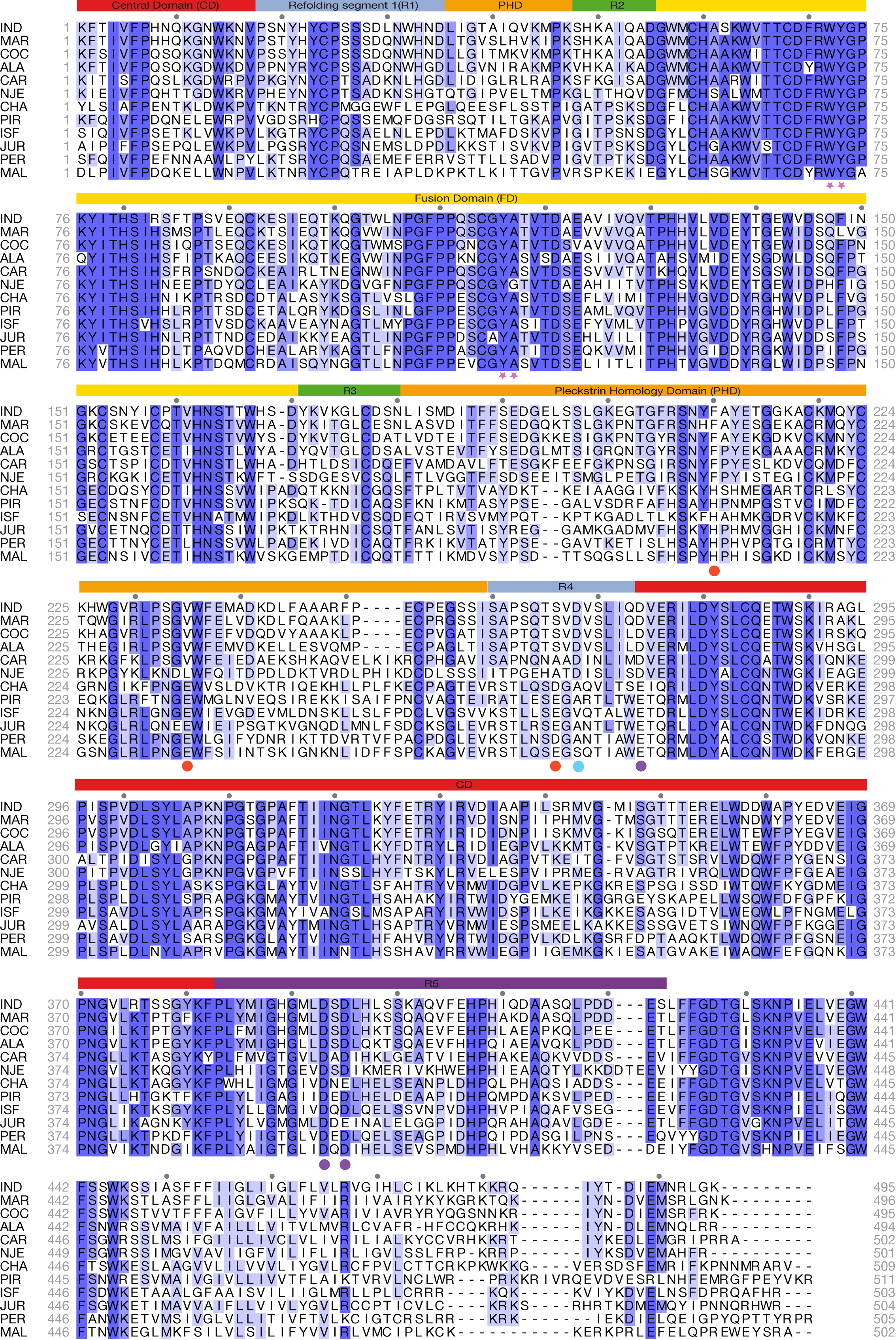

Supplement: S5 Fig — Conserved residues are highlighted in shades of blue. The black dashes show gaps. The colored bars above the sequences indicate the domains described for CHAV-G and VSVIND-G. Residues located in the fusion loops are indicated by pink stars below the sequences. Residues involved in the formation of the pH sensitive molecular switches are indicated by dots below the sequences (in red are the residues making the switch which is specific of CHAV-G, in cyan is the residue making the switch which is specific of VSV-G, in purple are the residues contributing to pH sensing and common to both glycoproteins [25]). Vesicular Stomatitis Indiana Virus (IND), Maraba Virus (MAR), Cocal Virus (COC), Vesicular Stomatitis Alagoas Virus (ALA), Carajas Virus (CAR), Vesicular Stomatitis New Jersey Virus (NJE), Chandipura Virus (CHA), Piry Virus (PIR), Isfahan Virus (ISF), Jurona Virus (JUR), Perinet Virus (PER), Malpais Spring Virus (MAL). The accession number for all these sequences is given in Fig. 6. (TIF) [file ppat.1004756.s005.tif]
